# The CD146-HIF-1α axis regulates epithelial cell migration and alveolar maturation in a mouse model of bronchopulmonary dysplasia

**DOI:** 10.1038/s41374-022-00773-z

**Published:** 2022-03-19

**Authors:** Rui Jin, Qianqian Gao, Chunyu Yin, Mengjia Zou, Keyu Lu, Wei Liu, Yuting Zhu, Mingshun Zhang, Rui Cheng

**Affiliations:** 1 Department of Neonatal Medical Center, Children’s Hospital of Nanjing Medical University, Nanjing, China; 2 Department of Neonatal Medical Center, Lianyungang Maternal and Child Health Hospital, Lianyungang, China; 3 Jiangsu Province Engineering Research Center of Antibody Drug, Nanjing Medical University, Nanjing, China; 4 NHC Key Laboratory of Antibody Technique, Department of Immunology, Nanjing Medical University, Nanjing, China; 5 Department of Neonatology, The Affiliated Wuxi Children’s Hospital of Nanjing Medical University, Wuxi, China

**Keywords:** Experimental models of disease, Integrin signalling

## Abstract

Bronchopulmonary dysplasia (BPD) is the most common challenge in preterm neonates. Retardation of alveolar development characterizes the pulmonary pathology in BPD. In the present study, we explored the roles of the CD146-HIF-1α axis in BPD. We demonstrated that the levels of reactive oxygen species (ROS) and soluble CD146 (sCD1146) were increased in the peripheral blood of preterm neonates with BPD. In alveolar epithelial cells, hyperoxia promoted the expression of HIF-1α and CD146, which reinforced each other. In a mouse model of BPD, by exposing pups to 65% hyperoxia, HIF-1α and CD146 were increased in the pulmonary tissues. Mechanistically, CD146 hindered the migration of alveolar epithelial cells; in contrast, movement was significantly enhanced in CD146-knockout alveolar epithelial cells. As expected, CD146-knockout ameliorated alveolarization and improved BPD disease severity. Taken together, our findings imply that the CD146-HIF-1α axis contributes to alveolarization and that CD146 may be a novel candidate in BPD therapy.

## Abstract (web summary)

Bronchopulmonary dysplasia (BPD) is still the most common challenge in preterm neonates. The authors found that reactive oxygen species and sCD146 are increased in preterm peripheral blood samples. They then show that the CD146-HIF-1α axis contributes to alveolarization and propose that CD146 may be a novel candidate in BPD therapy.

## Introduction

Bronchopulmonary dysplasia (BPD) in preterm neonates is a serious lung condition characterized by the retarded development of alveoli and vasculature^1^. Previously, we demonstrated that the extracellular matrix (ECM) protein fibronectin was involved in alveolar maturation and therefore contributed to the pathogenesis of BPD^2,3^. The maturation of alveoli largely depends on the behavior of alveolar epithelial cells, i.e., cell migration^4^. The ECM provides a scaffold for and guides alveolar epithelial cell migration^5^. As an adhesion molecule, CD146 regulates the migration of monocytes^6^ and endothelial cells^7,8^. In pulmonary epithelial cells, CD146 promotes the adherence of viruses^9^, bacteria^10^, and fungi^11^ to the pulmonary epithelium. However, the roles of CD146 in the migration of alveolar epithelial cells and the maturation of alveoli in the development of BPD are largely elusive.

With oxygen supplementation therapy, reactive oxygen species (ROS) are elevated in preterm infants, which is associated with impaired lung development^12^. In the endothelia cells, ROS burst induces CD146 dimerization, leading to angiogenesis^13^. In addition to CD146 dimer, CD146 may be bound with various receptors. Among of them, S100A8/A9 interacting with CD146 promotes the formation of ROS in melanoma cells^14^, suggesting that ROS and CD146 may be synergistic. Hypoxia-inducible factor 1α (HIF-1α), a pivotal player in the pathogenesis of BPD, may be potentially induced by ROS^15^. In the immature fetal lung, HIF-1α in the branching epithelium improves lung growth^16^. In postnatal lung developing, HIF-1α similarly increases vasculature growth and alveolar maturation^17^. As expected, HIF-1α stabilization preserves alveolar and vascular growth in the BPD model^18^. In contrast, HIF-1α deficiency reduces lung morphogenesis^19^. Although HIF-1α is required for lung growth, excessive HIF-1α, as the other side of the same coin, is deleterious to alveolar maturation^20^, suggesting that balanced production of HIF-1α should be strictly controlled in lung development. In endothelial cells, HIF-1α and CD146 reinforce each other, thereby promoting vasculature development^21^. Whether HIF-1α and CD146 mediate the epithelial cells migration in the BPD is unknown. In the present study, we aim to explore the hypothesis that HIF-1α and CD146 mediate alveolar epithelial cells migration and contribute to the development of BPD. Our data demonstrate that CD146 stimulated by ROS and HIF-1α suppress the migration of alveolar epithelial cells. CD146 deficiency alleviates the BPD disease severity.

## Materials and methods

### Preterm infants

Preterm infants born at gestational age ≤32 weeks were admitted to the neonatal intensive care unit at Children’s Hospital of Nanjing Medical University and were enrolled in the study between July 2020 and January 2021. The infants were diagnosed with BPD according to the workshop definition by the National Institutes of Child Health, the Human Development/National Heart, Lung, and Blood Institute and the Office of Rare Diseases^22^. Preterm infants without BPD were randomly selected for the control group. Infants with severe congenital malformations and inherited metabolic diseases were excluded from the study.

### ROS and sCD146 detection in plasma samples

Routine clinical data were collected for all enrolled infants. A total of 1 ml of fasting peripheral venous blood samples was taken and saved in an anticoagulant tube each time. The level of ROS was measured with a microplate reader (Biotek Instruments, Inc., USA) at an excitation wavelength of 488 nm and an emission wavelength of 525 nm. ROS were also captured by an Olympus IX73 inverted microscope at ×200 magnification. All ROS tests were performed in accordance with the manufacturer’s instructions (s0033, Beyotime, China). Soluble CD146 (sCD146) in human plasma was measured using a commercial sCD146 ELISA kit (E-EL-H2403c, Elabscience, China) according to the instructions provided by the manufacturer.

### Animals and ethics statement

Wild-type C57BL/6J mice (female and male) at 6–8 weeks of age were obtained from the Laboratory Animal Center of Nanjing Medical University (Nanjing, China) and bred under specific pathogen-free conditions at Nanjing Medical University. CD146-knockout (KO) mice on a C57BL/6J background were obtained from Cyagen, Suzhou, China.

### Animal model of BPD

The mouse model of BPD was established as described previously^23,24^. Briefly, newborn C57BL/6J pups from several litters were mixed and randomly divided into a control group and an experimental group. Then, the control group and the experimental group were exposed to indoor air (21% oxygen) or 65% oxygen within 24 h after birth until the 5th day, when the puppies exposed to oxygen returned to indoor air. The temperature (22 °C) and humidity (50–60%) were kept constant. To avoid oxygen toxicity in the dams and to eliminate maternal effects between groups, the nursing dams were rotated between their hyperoxia and room air litters every 24 h. All mice were maintained on a 12-h light-dark cycle. Mice were euthanized on 14 by the injection of pentobarbital sodium (200 mg/kg i.p.).

To explore the roles of CD146 in BPD pathogenesis, CD146-knockout (CD146 KO) pups were treated with 65% oxygen to establish a BPD model. Wild-type BPD mice was set as the control group.

### Lung sample preparation

Mice were sacrificed by intraperitoneal injection of sodium pentobarbital. After aortic transection, a thoracotomy was performed, the right bronchus was ligated, and the right lungs were removed and snap frozen. The tracheas were cannulated, and the left lungs were inflated and fixed with 4% paraformaldehyde (G1101, Servicebio, China) at a pressure of 25 cm H_2_O for ≥15 min. After equilibration, the left lungs were removed and fixed in 4% formalin overnight. Subsequently, paraffin embedded lung tissues were cut into 5 μm-thick sections on a Leica model 2165 rotary microtome (Leica, Nussloch, Germany) and stained with H&E as previously described^25,26^.

### H&E staining

Tissue sections were stained with hematoxylin and eosin (H&E) for morphometric analyses. To assess uniform and proportional samples from each lung, three nonoverlapping photomicrographs in different sections were captured at ×200 magnification with a microscope (model BX-53, Olympus Optical) under identical lighting conditions and optical settings by an investigator blinded to the grouping. Three images per animal were analyzed and averaged using research-based digital image analysis software (ImageJ, JAVA). The analytical methods of radial alveolar counts (RAC) and mean linear intercept (MLI) were determined by standard morphometric techniques^27,28^. Briefly, MLI was determined by superimposing a predetermined grid on the image, with set randomly placed lines, and the number of times the lines cross an air–tissue interface counted^28^. And alveolarization was assessed by standard RAC methods, as previously described^29,30^. Briefly, respiratory bronchioles were identified as bronchioles lined by epithelium in one part of the wall. From the center of the respiratory bronchiole, a perpendicular line was dropped to the edge of the acinus (connective tissues or septum or pleura), and the number of septae intersected by this line was counted.

### Immunohistochemical staining

In the fixed lung tissues, 5-μm sections were cut and deparaffinized. Antigen retrieval was performed in 10 mM citrate buffer, pH 6.0, in a pressure cooker for 10 min. Endogenous peroxidase activity was inhibited using a 3% H_2_O_2_ solution applied to the slides for 15 min, followed by a 30-min blocking step using 3% BSA in PBS. The slides were then incubated with rabbit polyclonal HIF-1α antibody (1:100, Servicebio, China) or rabbit monoclonal CD146 (1:200, ab75769, USA) for 1 h at room temperature. The slides were further stained with horseradish peroxidase (HRP)-conjugated goat anti-rabbit IgG (EarthOx Life Sciences, CA, USA) for 50 min at room temperature. Then, freshly prepared DAB chromogenic reagent was added to mark the tissues. Finally, the sections were counterstained with hematoxylin staining solution and captured at ×400 magnification with a microscope (model BX-53, Olympus Optical).

### Lung mechanics

As previously described^31^, the mouse trachea was exposed and connected to a computer-controlled small animal mechanical ventilator (flexiVent, SCIREQ). After two recruitment breathes of sustained inspiration up to a pressure of 30 cm H_2_O for 3 s, saline control and methacholine at 3.125, 6.25, 12.5, 25, 50, and 100 mg/ml (Sigma Aldrich) were aerosolized using an ultrasonic nebulizer diverted into the ventilator’s inspiratory flow over 10 s. Measurements of average respiratory system resistance (Rrs) and compliance (Crs) were made every 40 s over 5 min after each dose.

### Cell culture

Primary alveolar epithelial cells from mice were purified using 0.1% collagenase, 0.25% trypsin, and DNase I and were selected with mouse IgG (36111ES60, Yeasen, China) as previously described^32^. The MLE-12 cell line was obtained from ATCC (CRL-2110, USA) and cultured according to the protocol provided by ATCC. MLE-12 cells were cultured in DMEM (HyClone, USA) with 10% fetal bovine serum (Lonsera, USA) and 1% penicillin/streptomycin (HyClone, USA) in a humidified atmosphere of 5% CO_2_ at 37 °C overnight. MLE-12 cells were treated with 0, 50, 100, 250, or 500 μmol/l H_2_O_2_ or an equal volume of DMEM (HyClone, USA) as a control. When needed, the PI3K inhibitor LY294002 (MCE, USA, HY-10108) or ERK inhibitor U0126 (MCE, USA, HY-1203) was added to the medium at a final concentration of 10 µM. Cells were harvested 12 h later for further analysis.

### Cell transfection

MLE-12 cells were seeded and incubated overnight before transfection. After mixing with Liposomal Transfection Reagent (Yeasen, China) in DMEM without FBS, penicillin or streptomycin for 25 min, the expression plasmid encoding CD146 or HIF-1α (Abmgood, China) was then transfected into MLE-12 cells at 90–95% density in DMEM for 48 h. The cells were harvested for further analysis. The blank vehicle plasmid was set as a control.

### RNA isolation and quantitative real-time PCR

Total RNA was obtained from fresh lung tissue with a TRIzol reagent kit (Life Technologies) and reverse transcribed into cDNA with a reverse transcription kit (Abmgood, Zhenjiang, China) according to the manufacturer’s instructions. HIF-1**α**, CD146, and β-actin mRNA expression was quantified with a StepOnePlus Real-Time PCR System (ABI, USA). The primers used for real-time PCR were designed by referring to PrimerBank (https://pga.mgh.harvard.edu/primerbank). Primers with the following sequences were used: CD146 forward, 5-GGAAAATCAGTATCTGCCTCTCC-3; CD146 reverse, 5-GGAAAATCAGTATCTGCCTCTCC-3; HIF-1**α** forward, 5-ACCTTCATCGGAAACTCCAAAG-3; HIF-1**α** reverse, 5-CTGTTAGGCTGGGAAAAGTTAGG-3; β-actin forward, 5- GGCTGTATTCCCCTCCATCG-3; and β-actin reverse, 5-CCAGTTGGTAACAATGCCATGT-3. Relative levels were determined using the comparative CT method, and β-actin was used as the internal control^33^. Each sample was run in triplicate, and the results are representative of at least three independent experiments.

### Protein extraction and western blotting

Total protein was extracted from cells or tissues by lysis with RIPA buffer containing protease and phosphatase inhibitor cocktails (Beyotime, Shanghai, China) and sonicated on ice 3 times for 20 s each time. Protein concentrations were determined with a bicinchoninic acid (BCA) assay. Equal amounts of proteins were used for SDS–PAGE. The proteins were separated by 10% SDS–PAGE and transferred to polyvinylidene fluoride (PVDF) membranes (Millipore, Billerica, USA). The membranes were blocked for 1 h in 5% skim milk at room temperature and incubated at 4 °C overnight with the following primary antibodies: anti-CD146 (ab75769, Abcam), anti-HIF-1α (ab54385, Abcam), anti-Erk (4695, Cell Signaling Technology), anti-p-Erk (4370, Cell Signaling Technology), anti-Akt (4691, Cell Signaling Technology), anti-p-Akt (4060, Cell Signaling Technology) and anti-β-actin (4970L, Cell Signaling Technology). The membranes were then washed three times with Tris-buffered saline Tween-20 (TBST) and incubated with horseradish peroxidase (HRP)-conjugated goat anti-rabbit IgG (EarthOx Life Sciences, CA, USA) or goat anti-mouse IgG (H + L) HRP (s0002, Affinity Biosciences) for 1 h at room temperature. β-actin was used as an internal control. The antibody–antigen complexes were detected with Immobilon Western Chemiluminescent HRP Substrate (Millipore, MA, USA) and visualized using the G:Box gel doc system (Syngene, UK).

### Cell scratch test

When the cells seeded in 6-well plates reached a confluent state, a single scratch was made using a sterile yellow pipette tip. MlE-12 cells with normal or overexpression of CD146 were then incubated with FBS-free culture medium (to exclude the potential effects of FBS on cell migration) alone. Images of the scratches were captured at 0, 6, 12, and 24 h with an Olympus IX73 inverted microscope at ×200 magnification. The wound area was examined by the wound margin and calculated using ImageJ^34^. Cell mobility = (scratch width at 0 h − scratch width after culture)/scratch width at 0 h × 100%.

### Transwell migration assay

Transwell migration assays were performed using Transwell inserts (Corning Costar, USA) with a filter of 6.5 μm pores. Primary alveolar epithelial cells (WT or CD146 KO) were obtained according to a previous method^32^.  A total of 1 × 10^4^ cells in serum-free medium were seeded into the upper chamber of the insert, and complete medium was added to the lower chamber. After 24 h of incubation, the cells were fixed with 4% paraformaldehyde (G1101, Servicebio, China) and stained with Giemsa. Then, cells on the top surface of the membrane were wiped off, and cells on the lower surface were examined under an Olympus BX-53 microscope at ×200 magnification. Five random fields were photographed for counting purposes, and the average number of migrated cells was used as a measure of migration capacity.

### Live-cell imaging technique

Primary alveolar epithelial cells (1 × 10^3^/ml, WT or CD146 KO) were seeded onto a 96-well cell culture plate and treated with complete medium. Then, the 96-well cell culture plate was transferred into the temperature- and CO_2_-controlled (37 °C, 5% CO_2_) environment of a Zeiss Cell Discoverer microscope system. Live-cell phase-gradient contrast images of the individual field regions inside each well were automatically acquired using ZEN Blue 2.3 software. Representative figure images were selected, and additional image postprocessing steps were performed in ImageJ.

### Statistical analysis

Statistical analyses were performed using GraphPad Prism 8.0 software. Values are expressed as the means ± SDs. Differences between the groups were assessed by one-way analysis of variance and the Student–Newman–Keuls test (for multiple comparisons). Statistical significance was defined as follows: *P < 0.05; **P < 0.01; ***P < 0.001; ****P < 0.0001, and NS, not significant.

## Results

### Increased ROS and sCD146 in peripheral blood from BPD infants

**Fig. 1:**
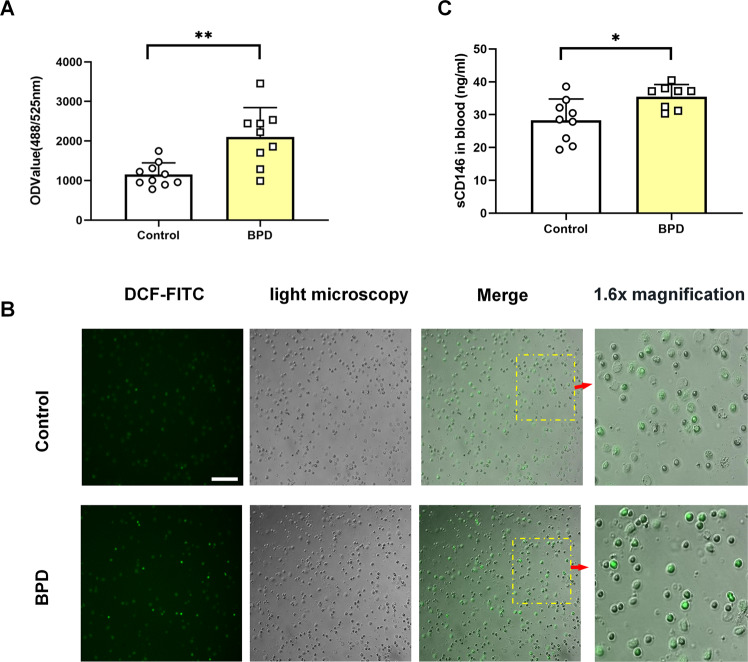
Increased ROS and sCD146 in the peripheral blood of BPD infants. Venous blood samples were taken at 4 weeks, and ROS in whole blood cells (except red blood cells) and plasma sCD146 were quantified. **A**, **B** ROS were significantly increased in the peripheral blood of BPD patients. **C** sCD146 was notably increased in the plasma from BPD patients. *P < 0.05; **P < 0.01; ***P < 0.001. Of note, due to the limited volume in each blood sample, only nine samples after ROS quantification were included for sCD146 assay.

In preterm infants, supplemental oxygen is frequently needed, which adds to the risk of oxidant injury^35^. Accordingly, to investigate the effects of oxidant injury on preterm infants, we recruited 19 preterm infants, including 9 BPD infants and 10 non-BPD control neonates (Table 1). Compared with the control group, ROS levels in the peripheral blood from BPD infants were significantly increased (Fig. 1A, B). Additionally, we found that plasma sCD146 was concurrently increased in BPD neonates (Fig. 1C). In sum, ROS and CD146 were increased in preterm neonates with BPD, which may play important roles in the pathogenesis of BPD.

**Table 1:** Clinical characteristic of the BPD and non-BPD infants.

Clinical features	non-BPD (*n* = 10)	BPD (*n* = 9)	*T*/*χ*2 value	*P* value
Birth weight (grams)	1.18 ± 0.42	0.99 ± 0.26	1.169^a^	0.258
Gestational age (days)	206.3 ± 17.89	194.67 ± 12.5	1.626^a^	0.122
Male gender	5 (50.0%)	5 (55.6%)	–	1.000
Apgar 1 min	8 ± 0.81	5 ± 2.83	3.218^a^	0.005
Apgar 5 min	8.5 ± 1.27	6.9 ± 1.69	2.364^a^	0.030
CPAP (days)	3.8 ± 3.08	19.4 ± 7.94	−5.547^a^	0.000
Mechanical ventilation (days)	1.8 ± 3.55	25.3 ± 18.89	−3.877^a^	0.001
Days with oxygen	13.50 ± 9.372	73.3 ± 30.104	−5.988^a^	0.000
Intraventricular hemorrhage (IVH)	9 (90%)	9 (100%)	–	1.00
Periventricular leukomalacia (PVL)	0 (0%)	0 (0%)	–	–
Necrotizing enterocolitis (NEC)	3 (30.0%)	3 (33.3%)	–	1.00
Late-onset neonatal sepsis (LOS)	1 (10.0%)	2 (22.2%)	–	0.582
Surfactant treatment	2 (20.0%)	7 (77.8%)	–	0.023
Patent ductus arteriosus (PDA)	7 (70%)	8 (88.9%)	–	0.582

*–* no chi-square value.

*P* value is calculated by fisher’s exact probability method.

^a^Is T value.

### ROS directly induced HIF-1α expression in epithelial cells

**Fig. 2:**
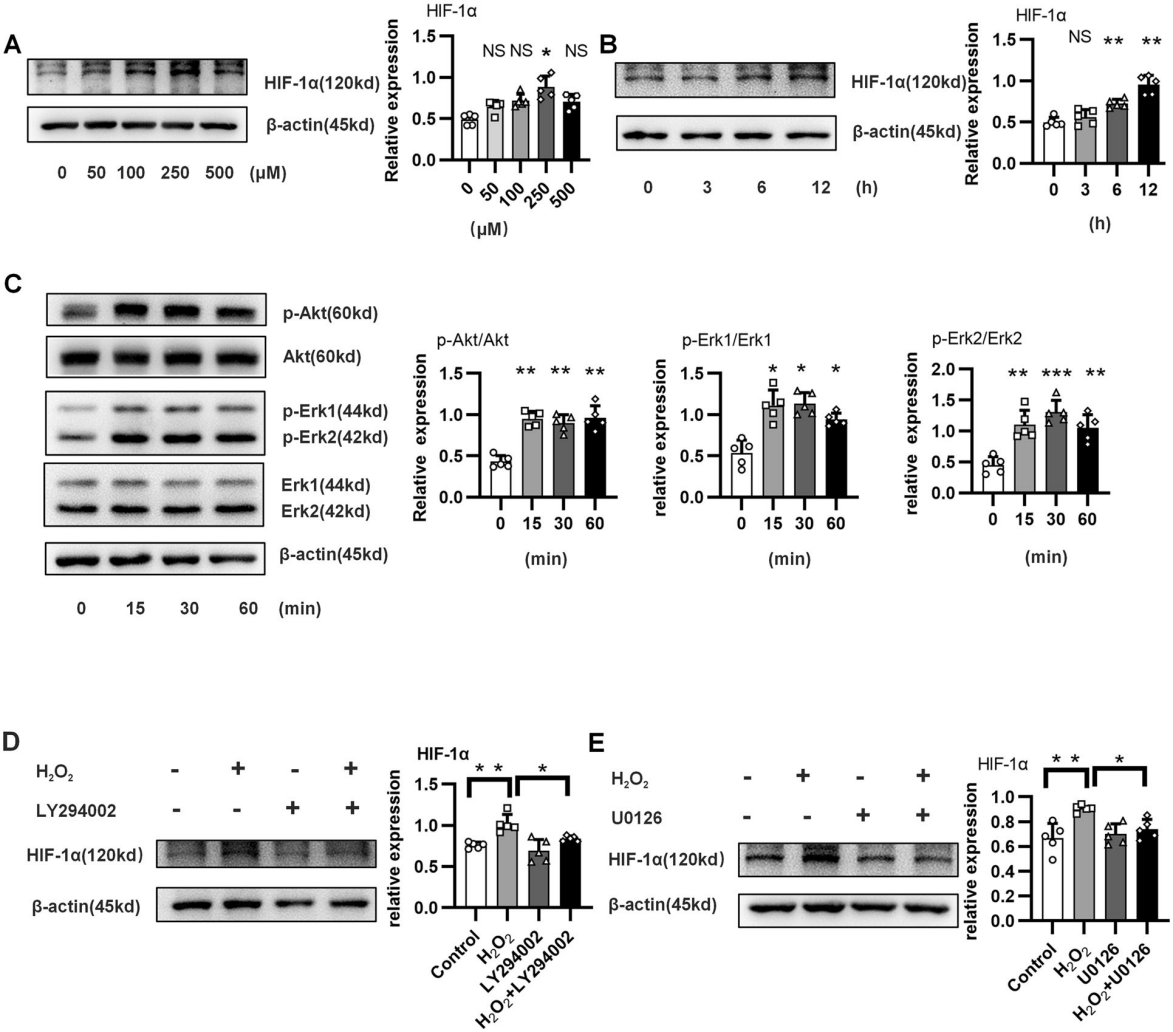
ROS directly induced HIF-1α expression in epithelial cells. **A** Dose-dependent expression of the HIF-1α protein in MLE-12 cells stimulated with H_2_O_2_. **B** Time-dependent expression of the HIF-1α protein in MLE-12 cells stimulated with 250 μM H_2_O_2_. **C** Erk and Akt protein expression in MLE-12 cells stimulated with 250 μM H_2_O_2_ for 12 h. **D**, **E** HIF-1α protein expression in MLE-12 cells stimulated with 250 μM H_2_O_2_ for 12 h in the presence or absence of the ERK inhibitor U0126 or the PI3K inhibitor LY294002. All experiments were repeated at least 3 times. *P < 0.05; **P < 0.01; ***P < 0.001.

Hyperoxia can lead to excessive production of ROS^36^, which not only promotes the transcription of HIF-1α^37^ but also maintains the stability of HIF-1α^38^. To simulate hyperoxia-induced lung injury, alveolar epithelial cells were treated with H_2_O_2_. As shown in Fig. 2A, B, H_2_O_2_ induced the expression of HIF-1α in epithelial cells. Previous studies have shown that the ERK pathway and PI3K pathway play important roles in ROS-induced HIF-1α expression^39–41^. Unsurprisingly, H_2_O_2_ activated the ERK and PI3K/Akt pathways in alveolar epithelial cells (Fig. 2C). Furthermore, inhibitors of ERK (U0126) and PI3K (LY294002) rescued the expression of HIF-1α in epithelial cells treated with H_2_O_2_ (Fig. 2D, E). Overall, these results indicated that H_2_O_2_ directly promoted the expression of HIF-1α in lung epithelial cells through the ERK and PI3K/Akt pathways.

### Hyperoxia-induced HIF-1α and CD146 expression in epithelial cells

**Fig. 3:**
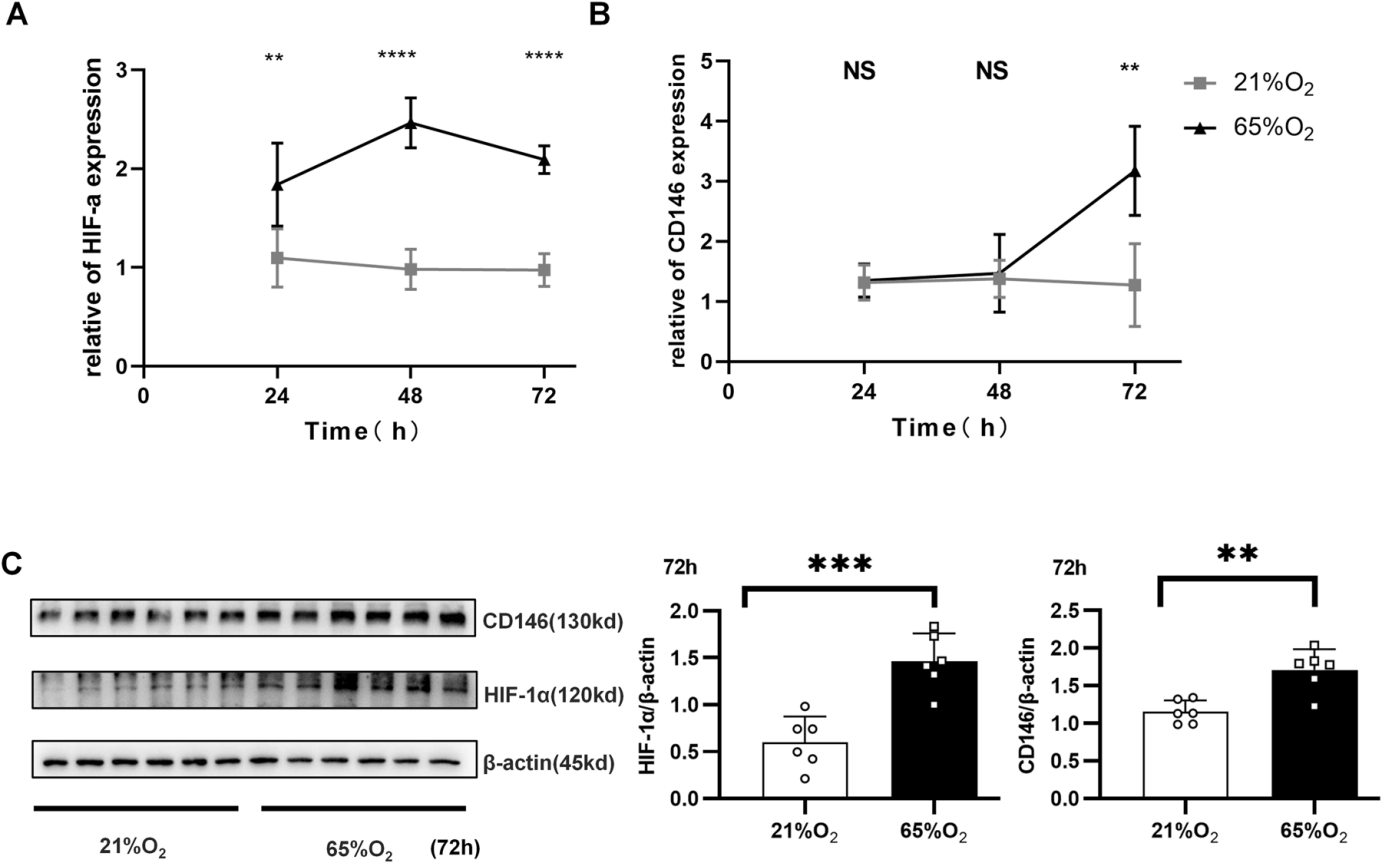
Hyperoxia directly induced hif-1α and CD146 expression in epithelial cells. **A** The expression of HIF-1α mRNA in MLE-12 cells treated with 65% O_2_ for 24, 48, and 72 h was detected by qRT–PCR. **B** The expression of HIF-1 mRNA in MLE-12 cells treated with 65% O_2_ for 24, 48, and 72 h was detected by qRT–PCR. **C** HIF-1α and CD146 protein expression in MLE-12 cells stimulated with 65% O_2_ at 72 h. All experiments were repeated at least 3 times. *P < 0.05; **P < 0.01; ***P < 0.001.

To better simulate oxygen-induced lung injury in the clinic, we directly treated lung epithelial cells with hyperoxia (65% oxygen). At the gene level, 65% hyperoxia significantly increased HIF-1α in alveolar epithelial cells at 24 h (Fig. 3A), but CD146 expression was upregulated until 72 h post hyperoxia (Fig. 3B). Similarly, the protein levels of CD146 and HIF-1α in alveolar epithelial cells were significantly increased after 72 h of stimulation with 65% oxygen (Fig. 3C). Together, these results indicated that hyperoxia directly induced the expression of HIF-1α and CD146 in alveolar epithelial cells.

### Positive feedback of HIF-1α and CD146 in epithelial cells

**Fig. 4:**
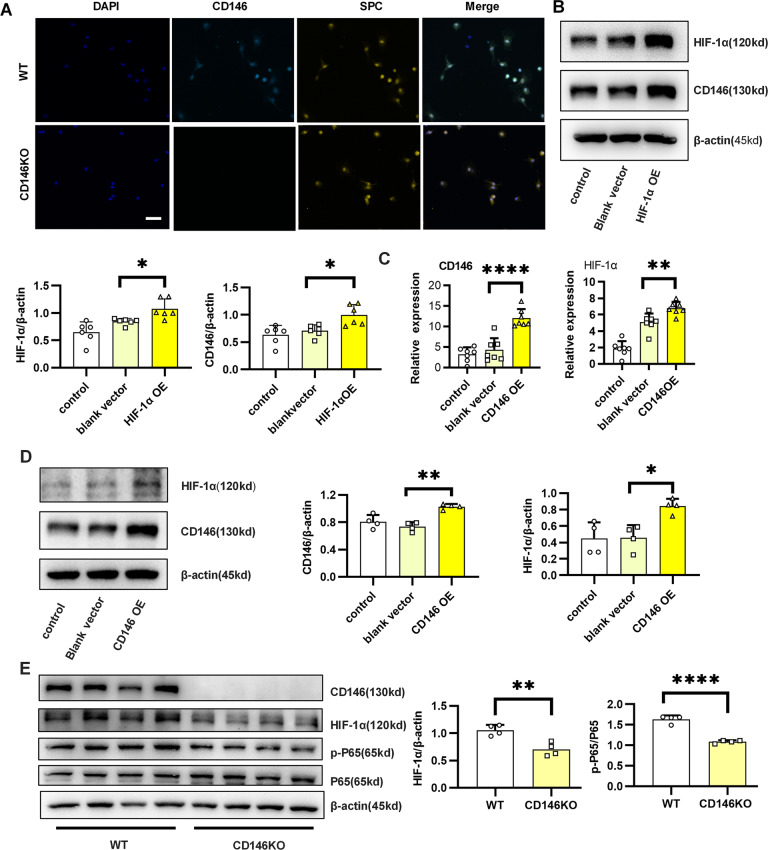
Positive feedback of HIF-1α and CD146 in epithelial cells. **A** Representative immunofluorescence imaging of CD146 in WT or CD146 KO primary alveolar epithelial cells. Scale bars, 200 μm. **B** Western blot analysis of HIF-1α and CD146 expression in MLE-12 cells treated with a HIF-1α expression plasmid. **C**, **D** qRT–PCR and Western blot analysis of HIF-1α and CD146 expression in MLE-12 cells treated with a CD146 expression plasmid. **E** Western blot analysis of CD146, HIF-1α, and P65 expression in primary alveolar epithelial cells. All experiments were repeated at least 3 times. *P < 0.05; **P < 0.01; ***P < 0.001.

Thus far, we demonstrated that ROS and sCD146 were increased in BPD patients and that ROS promoted HIF-1α expression. Therefore, we further explored the potential relationships between HIF-1α and CD146. As shown in Fig. 4A, SPC-positive alveolar epithelial cells co-expressed CD146. We then overexpressed HIF-1α in the alveolar epithelial cell line MLE-12. Accompanied by HIF-1α elevation, CD146 was significantly increased (Fig. 4B). This result was consistent with previous studies showing that HIF-1α binding to CD146 hypoxia response element (HRE) promoted CD146 transcription^42^.

Given that CD146 promoted the expression of NF-κB (P65)^43,44^ and P65 regulated HIF-1α expression^45^, we hypothesized that CD146 may be involved in the regulation of HIF-1α expression. To explore this, we overexpressed CD146 in MLE-12 cells and found that HIF-1α was upregulated in CD146-overexpressing epithelial cells (Fig. 4C, D). Similarly, the expression of pP65/P65 and HIF-1α was notably decreased after CD146 knockout (Fig. 4E). In short, these results suggested that the CD146-HIF-1α axis formed a positive feedback loop in alveolar epithelial cells.

### Increased pulmonary HIF-1α and CD146 in a mouse model of BPD

**Fig. 5:**
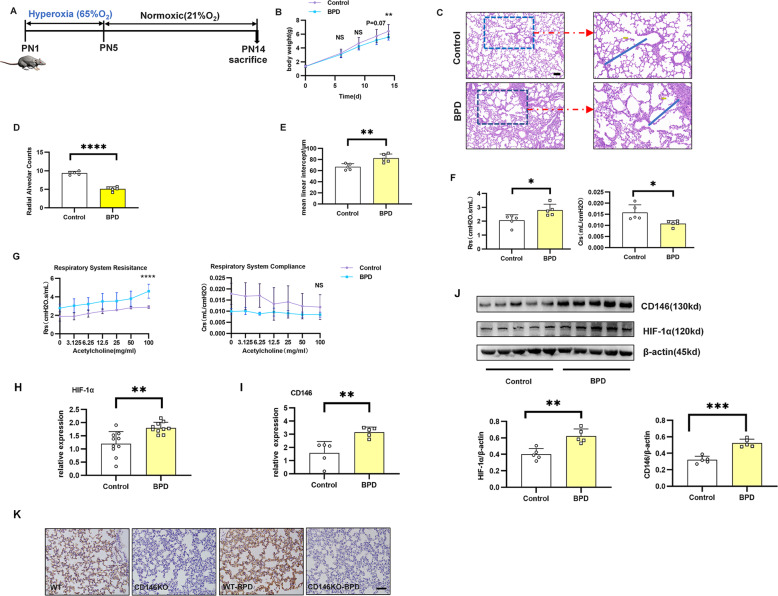
Increased HIF-1α and CD146 in the early stage of BPD. Newborn mice were exposed to 65% hyperoxia until the 5th day, when the puppies exposed to oxygen returned to indoor air to establish the BPD model. **A** Flow chart showing the BPD model. **B** Body weight was recorded at 6, 9, 12, and 14 days after birth. **C**–**E** Lung morphometry was analyzed by H&E staining of tissue samples from surviving pups at day 14 post birth. Scale bars = 100 μm. **F** Determination of respiratory resistance (Rrs) and compliance (Crs) in the lungs of BPD and normal mice by the finepointerc system. **G** Methacholine dose-dependent changes in Rrs and Crs were observed in BPD and control mice. **H**, **I** qRT–PCR and Western blot analysis of HIF-1α and CD146 expression in pulmonary tissues at day 14 post birth. **J**, **K** Representative results of immunohistochemical analysis of CD146 in pulmonary tissues at day 14 post birth. Five mice were included in the control and BPD groups at each time point. All experiments were repeated at least 3 times. Scale bars, 100 μm. *P < 0.05; **P < 0.01; ***P < 0.001.

To demonstrate the significance of the CD146-HIF-1α axis in BPD, we established a murine model of BPD with postnatal hyperoxia. As depicted in Fig. 5A, pups were exposed to either indoor air (21% oxygen, control group) or 65% oxygen (BPD group) until the 5th day. The hyperoxia-treated mice weighed an average of 16.2% less at P14 than the control mice reared under normoxic conditions (Fig. 5B), as reported in a previous publication^46^. Compared with the control group, histological analysis demonstrated arrested alveolar development, with a significantly decreased number of large, simplified alveoli in the BPD group. In contrast, the mean linear intercept in the BPD group was significantly increased (Fig. 5C–E). In addition, the BPD group also showed notably increased respiratory resistance (Rrs) and decreased compliance resistance (Crs) (Fig. 5F). After inhalation of acetylcholine, the BPD group displayed significantly increased Rrs and slightly decreased Crs (Fig. 5G).

To explore whether the CD146-HIF1-α axis was involved in the pathogenesis of BPD, we measured the expression of CD146 and HIF-1α in pulmonary tissues using PCR and Western blot, and both HIF-1α and CD146 were significantly higher in the BPD group than in the control group (Fig. 5H–J). Moreover, immunohistochemistry assays (Fig. 5K) validated that CD146 was more pronounced in the BPD group. Collectively, these results implied that the CD146-HIF-1α axis was notably important at the early stage of BPD disease.

### CD146 decelerated the alveolar epithelial cells

**Fig. 6:**
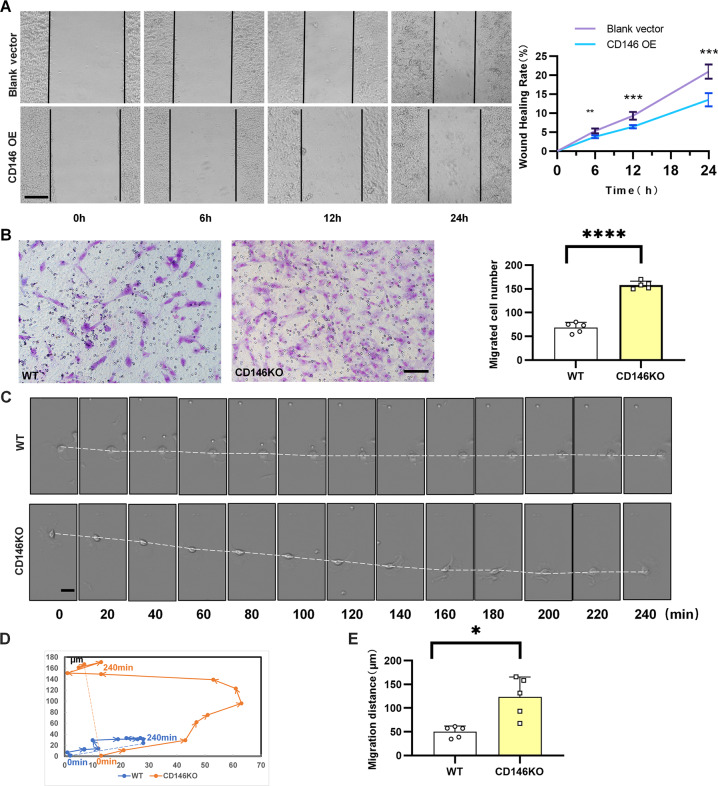
CD146 decelerated alveolar epithelial cells. **A** The migration ability of alveolar epithelial cells was measured by the scratch method. **B** The migration ability of alveolar epithelial cells was measured by Transwell assay. **C** Time-lapse images of alveolar epithelial cells are shown every 20 min over 240 min. **D** Tracing the two cells for 2 h, lines with arrows represent the total migration distance, and arrows represent the direction of motion. Lines without arrows represent the relative migration distance of these two cells. **E** Migration distance was significantly longer in the CD146 KO group. All experiments were repeated at least 3 times. *P < 0.05; **P < 0.01; ***P < 0.001.

Preterm infants with BPD display deficits in alveolarization, cellular migration, and injury repair^47,48^. CD146 regulates cell migration in a variety of ways. CD146 inhibits migration in activated T cells^49^ and trophoblast cells^50^ but promotes migration in tumors^51^ and endothelial cells^7^. We hypothesized that CD146 may regulate alveolar epithelial cell migration and thereby participate in BPD pathogenesis. To assess this, a scratch wound migration assay, Transwell chamber migration assay, and live-cell imaging were performed. In the scratch wound migration assay (Fig. 6A), the migration distance of epithelial cells that had been transfected with CD146 was significantly reduced compared with that of the controls. Conversely, the migration number of CD146-knockout alveolar epithelial cells was notably increased compared with that of the controls in the Transwell chamber migration assay (Fig. 6B). Similarly, time-lapse imaging showed that the velocity of CD146-knockout alveolar epithelial cells was significantly increased compared with that of the WT controls (Fig. 6C–E). Together, these results suggested that CD146 decelerated alveolar epithelial cells; CD146 deficiency promoted the maturation of alveoli in the developing lung and therefore may ameliorate the severity of BPD.

### CD146 deficiency relieved disease severity of BPD

**Fig. 7:**
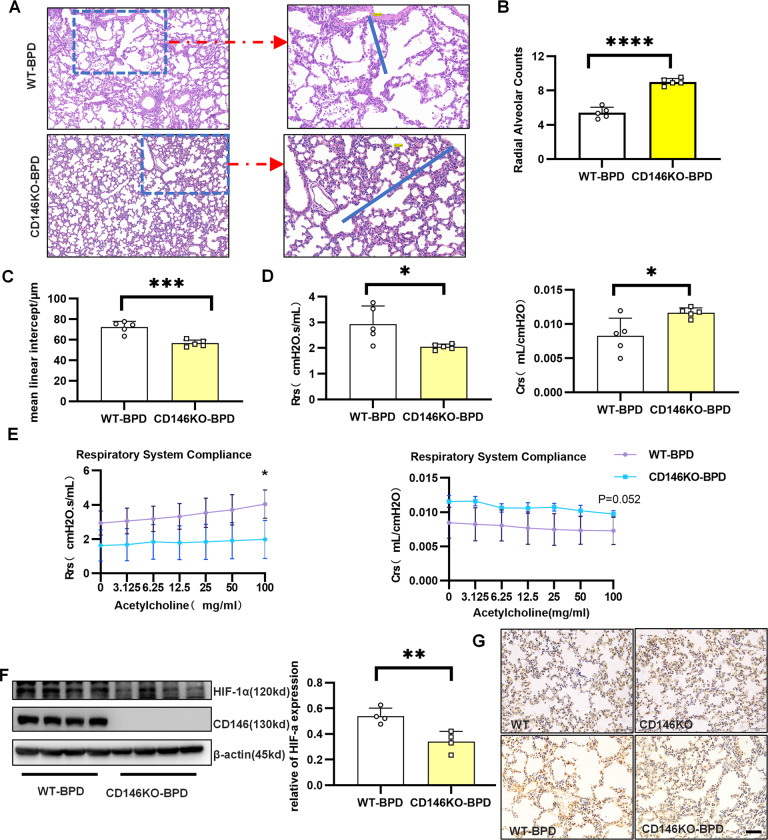
CD146 deficiency relieved the disease severity of BPD. Newborn mice were exposed to 65% hyperoxia until the 5th day, when the puppies exposed to oxygen returned to indoor air to establish the BPD model. **A**–**C** Lung morphometry was analyzed by H&E staining of tissue samples from surviving pups at day 14 post birth. Scale bar, 100 μm. **D** Determination of respiratory resistance (Rrs) and compliance (Crs) in the lungs of BPD mice by the finepointerc system. **E** Methacholine dose-dependent changes in Rrs and Crs were observed in BPD mice. **F** HIF-1α and CD146 protein expression in pulmonary tissues at day 14 post birth. **G** Representative results of immunohistochemical analysis of HIF-1α in pulmonary tissues at day 14 post birth. Scale bars, 200 μm. At least 3 mice were included in the control and BPD groups at each time point. All experiments were repeated at least 3 times. *P < 0.05; **P < 0.01; ***P < 0.001.

To provide direct genetic evidence that disrupting the CD146-HIF-1α axis may affect BPD development, we established a mouse model of BPD using CD146-knockout mice. As shown in Fig. 7A–C, histological analysis on day 14 post birth showed that CD146 deficiency improved alveolar development, with a significantly increased number of alveoli. Conversely, the mean linear intercept was remarkably diminished in CD146-deficient BPD-like mice. Similarly, CD146-deficient BPD-like mice showed notably decreased Rrs and increased Crs (Fig. 7D). After inhalation of acetylcholine, the CD146-deficient BPD-like mice also displayed a significant reduction in Rrs and an increase in Crs (Fig. 7E). Using Western blot assays (Fig. 7F) and immunohistochemistry (Fig. 7G), we discovered that HIF-1α was significantly lower in CD146-deficient BPD-like mice than in wild-type BPD-like mice at 14 days after birth. Collectively, these results implied that CD146 deficiency disrupting the CD146-HIF-1α axis at least partially alleviated BPD disease severity.

## Discussion

Despite escalating efforts in prevention, BPD incidence in preterm infants (<28 weeks gestational age) remains stable at 43%^52^. Retardation and simplification of lung alveolarization are the key histopathological characteristics in preterm infants with BPD. In the term mouse model of BPD^53^, lung development in pups (E17.5–PN5) resembled that in preterm infants (24–38 weeks gestational age). In the present study, we validated that ROS were elevated in the peripheral blood of BPD infants. Furthermore, in alveolar epithelial cells, hyperoxia increased HIF-1α and CD146, which reciprocally promoted each other. In a mouse model of BPD stimulated with 65% oxygen, HIF-1α and CD146 in the lung tissues were increased. CD146 deficiency promoted alveolar maturation and lung development; therefore, CD146 deficiency ameliorated the disease severity of BPD. RAC and MLI, the most used markers for lung morphometry in the BPD study^54,55^, were increased in the CD146 defective BPD-like mice. Of note, RAC and MLI may be biased; and the gold standard of lung morphometry is design-based stereology^56^. Nevertheless, lung functions (Rrs, Crs) were assayed in the present study, which to some extent overcome the pitfalls of RAC and MLI in the lung morphometry. Mechanistically, CD146 decreased the migration of alveolar epithelial cells, and CD146 deficiency, as expected, increased the migration of alveolar epithelial cells.

In line with a previous report that ROS levels were higher in the BPD group^57^, we evidenced that ROS levels in peripheral blood cells and plasma were higher in preterm neonates with BPD. Either H_2_O_2_ or 65% oxygen in the epithelial cell culture system simulated elevated ROS in BPD patients. As expected, H_2_O_2_ or 65% oxygen increased HIF-1α and CD146, which was at least partially dependent on the Erk and PI3K/Akt pathways. In pulmonary artery smooth muscle cells, HIF-1α and CD146 reinforce each other^58^. Similarly, the positive feedback loop of HIF-1α and CD146 was observed in alveolar epithelial cells. ROS increased HIF-1α, which subsequently promoted the expression of CD146 in epithelial cells. CD146 may activate the NF-κB signaling pathway, leading to the elevation of HIF-1α. Disruption of the HIF-1α and CD146 axes in CD146-deficient mice alleviated the disease severity of pulmonary arterial hypertension^58^. In a mouse model of BPD, CD146 deficiency mitigated the arrest of lung development. The vasculature system is vital in lung maturation. Pulmonary arterial hypertension is a serious complication of BPD^59^. Therefore, we could not preclude the possibility that the improved vascular remodeling in CD146-deficient mice may promote lung development and therefore provide relief from BPD.

Nonetheless, alveolar arrest is the key characteristic in BPD pathogenesis. In the preterm neonates with immature lung, oxygen therapy is a two-edged sword^60^. Airway epithelia cells exposed to oxygen and ROS may be damaged. In the alveoli, type 2 alveolar epithelial cells (AT2) rapidly migrate to the wound area and differentiate into type 1 alveolar epithelial cells (AT1)^61^. AT2 migration is regulated by various factors. Fibronectin in the ECM and its associated intergin directed the migration of alveolar epithelial cells^62^. The non-receptor tyrosine kinase focal adhesion kinase-1 (FAK) and the chemokine receptor CXCR4 also regulate the migration of alveolar epithelial cells in lung injury^63^. CD146 is a multifunctional molecule^64^. As in activated T cells^49^ and trophoblast cells^50^, CD146 suppressed migration in alveolar epithelial cells. Therefore, increased CD146 in the pulmonary tissues from BPD-like mice caused arrested alveolarization, and CD146 deficiency improved lung growth in BPD-like mice. CD146 may be a potential candidate in BPD therapy.

## Data Availability

The data that support the findings of this study are available on request from the corresponding author upon reasonable request.
